# Camp, Nursing, and the Politics of Seriousness

**DOI:** 10.1111/nup.70099

**Published:** 2026-07-16

**Authors:** Teresa A. Graziano

**Affiliations:** ^1^ University of Vermont Department of Nursing Burlington Vermont USA

**Keywords:** camp, nursing, nursing professionalism, queer nursing, queer theory, seriousness

## Abstract

This paper examines how camp, an aesthetic grounded in exaggeration, humor, and theatricality, functions as a legitimate nursing practice that challenges the cisheteronormative seriousness embedded in professional nursing culture. I begin by tracing how seriousness became a dominant aesthetic standard in nursing, shaping expectations for comportment, emotional restraint, and professionalism in ways that marginalize queer and trans nurses. Drawing from Sontag's *Notes on Camp*, I argue that seriousness is not an objective requirement of safe or ethical care but a culturally specific performance that restricts who and what count as ‘professional’. I then introduce camp as a counter‐aesthetic that exposes the performative nature of these norms. Through playfulness and intentional artifice, camp destabilizes the boundaries of professional conduct and opens space for alternative, culturally grounded expressions of care. To illustrate this, I examine the work of Bobbi Campbell, known as Sister Florence Nightmare RN, as a historical exemplar of a drag nurse who promoted health education, reduced stigma, and community resilience during the early HIV/AIDS epidemic. I then pull on Nurse Anne Thracks and Mandy Mango as contemporary examples of drag‐as‐nursing. These examples demonstrate how queer nurses use drag and camp to strategically engage communities, communicate health information, and model radically inclusive care. I extend this analysis to contemporary nursing contexts, arguing that integrating camp is a form of ethical authenticity aligned with the profession's commitments to dignity, self‐regard, and social justice. I show how campy and drag nurses actively subvert restrictive norms by transforming the figure of the nurse—through language, attire, and performance—while still providing effective, culturally relevant care. Ultimately, I propose camp as a generative framework for reimagining nursing aesthetics and practice. Embracing camp expands the boundaries of what is considered professional, ethical, and therapeutic, offering a model of nursing that honors joy, relationality, sociopolitical wellness.

It's 1982. A nun dressed in a habit is standing on a stage while the audience looks on. She is wearing a pair of white, geometric sunglasses that project from her face like butterfly wings. Her wrist is adorned with a silver men's watch, which matches the silver handcuffs on her hips. To add extra flair, she has a string of pearls draped around her neck. This nun has a black name tag reading “Sister Florence Nightmare RN [Real Nun].” She is presenting to onlookers at the 2nd Annual Dog Show and Parade, an early fundraiser for the San Francisco Kaposi's Sarcoma Cancer Clinic (Carhaix 1982). The man behind this drag persona is Bobbi Campbell, a public health nurse and aspiring nurse practitioner (White 1984), a gay man living with HIV/AIDS, and the sardonically self‐proclaimed ‘AIDS Poster Boy’ (France 2016; Lipsky 2021).

At first glance, Bobbi's appearance may seem incompatible with conventional expectations of nursing professionalism. Yet, his work as Sister Florence Nightmare RN demonstrates something more complicated and more politically significant than parody or spectacle. Through drag, theatricality, humor, and exaggeration, Bobbi educated gay men about sexually transmitted infections, reduced stigma surrounding HIV/AIDS, raised money for community care, and built trust within populations increasingly abandoned by mainstream healthcare systems. His performances were camp, but they were also unmistakably acts of nursing.

I argue throughout this paper that camp is not merely an aesthetic style or entertainment practice but can act as a culturally grounded model of nursing that disrupts cisheteronormative constructions of professionalism. I use ‘camp’ here not merely to describe flamboyance or humor, but to identify an aesthetic and political practice that uses exaggeration, theatricality, irony, and play to expose social norms as constructed rather than natural (Sontag 1964). Camp is not inherently liberatory, caring, or queer, nor is all nursing camp. Rather, I examine moments in which camp becomes mobilized in the service of nursing care through health education, advocacy, stigma reduction, trust‐building, and culturally resonant forms of relationality.

This distinction matters because nursing professionalism has historically been organized around ideals of seriousness, restraint, decorum, and moral respectability that are often treated as neutral or universal. In practice, however, these norms are deeply shaped by cisheteronormative, racialized, gendered, and classed expectations regarding which bodies, affects, and performances are considered professionally legitimate. For queer and trans nurses in particular, professional seriousness can function less as a neutral standard than as a disciplinary aesthetic that pressures them to suppress forms of expression, relationality, humor, embodiment, and cultural practices that fall outside dominant norms. Camp offers one way of exposing the constructed nature of these expectations while opening alternative possibilities for nursing practice rooted in authenticity, joy, and sociopolitical care.

This intervention feels especially urgent within the contemporary political climate. Across the United States, drag performance, queer embodiment, and trans visibility have become increasingly targeted through legislation, institutional regulation, and moral panic. Simultaneously, healthcare systems continue to struggle to provide culturally safe care for queer and trans communities who remain disproportionately vulnerable to discrimination, mistrust, and barriers to access. Against this backdrop, camp becomes more than aesthetic expression. It can function as a resistant mode of care that creates relational openings between nurses and communities historically harmed by healthcare institutions.

To develop this argument, I first define camp and clarify its relationship to drag, queer theory, and nursing aesthetics. I then examine how professionalism and seriousness operate within nursing as a culturally specific performance rather than neutral standards. From there, I turn to Bobbi's legacy as a Sister of Perpetual Indulgence as a historical example of camp functioning through health education, advocacy, and community care during the HIV/AIDS crisis. I then examine contemporary drag nurses, including Nurse Anne Thracks and Mandy Mango, to illustrate how camp functions therapeutically, pedagogically, and politically by fostering trust, reducing stigma, and cultivating queer joy. Finally, I propose that camp is not opposed to nursing practice, but instead offers a corrective to the restrictive politics of professionalism by expanding the ways ethical, authentic, and relational nursing care can look.

## 1 Reflexivity Statement

Understanding why camp matters in nursing requires acknowledging my standpoint. I am a feminist, white, queer, nonbinary/femme nurse who integrates camp into their nursing practice in clinical and educational settings. I engage with drag only as an enthusiastic spectator. This perspective shapes my reading of camp as a cultural practice within queer communities and informs my valuation of its role in critiquing and subverting cisheteronormative expectations of professionalism, emotional restraint, and respectability in nursing practice.

This standpoint also shapes how I engage tensions surrounding camp and drag. Some feminists, particularly those emerging from strands of second‐wave feminism, critique drag for grotesquely reproducing misogynistic caricatures of women through exaggerated femininity (Murphy 2014). These critiques often emerge from concerns that performers appropriate femininity as costumes while women remain materially constrained by gendered oppression. While these concerns deserve serious engagement, queer and trans scholarship has also demonstrated that drag and camp can expose gender itself as theatrical, stylized, and socially constructed rather than biologically fixed or naturally embodied. Drag and camp operate not by mocking women, but by destabilizing the assumption that any gender performance is ‘natural’ in the first place.

Importantly, camp and drag are not interchangeable. Drag refers to the performance of gender through stylization, exaggeration, theatricality, and embodiment (Butler 1999). Camp is a broader aesthetic and political sensibility that embraces artifice, excess, irony, humor, and performativity (Sontag 1964). Drag may enact camp, but camp can emerge outside formal drag performance through modes of speech, dress, relationality, humor, pedagogy, and care. Likewise, not all queer nursing is camp‐inflected, and not all camp functions ethically. This paper therefore does not argue that camp is inherently therapeutic or that all nurses should perform drag. Rather, I examine how camp can operate as a culturally meaningful and ethically resonant form of nursing intervention under particular social and historical conditions.

In what follows, I show how camp and drag might reimagine nursing aesthetics in ways that expand beyond the cisheteronormative framework the profession is often confined in. In doing so, camp nursing cultivates forms of holistic care that attend to physical wellbeing, along with sociopolitical wellness, dignity, community survival, and queer joy.

### 1.1 Camp, Drag, and Queer Aesthetics of Care

It is necessary to define what camp is and why it matters politically, aesthetically, and relationally. Camp is frequently associated with flamboyance, drag performance, or ironic humor, but it is more accurately understood as an interpretive sensibility and cultural practice that foregrounds exaggeration, theatricality, artifice, stylization, and excess (Sontag 1964). Sontag (1964) described camp as a mode of seeing the world ‘in quotation marks’, one that delights in performance, incongruity, and exaggeration while exposing the constructed nature of norms often mistaken for natural truths. Camp refuses authenticity as innocence, instead reveling in performance. Camp makes visible the labor involved in producing coherence, seriousness, and legitimacy.

Camp holds significance within queer communities because it offers tools for surviving and negotiating hostile social worlds. Historically, queer and trans people have often been required to navigate systems that render their identities deviant, pathological, or socially unintelligible. Camp emerged, in part, as a way of transforming marginalization into expressive practice. Humor, theatricality, irony, and exaggeration became mechanisms through which queer communities could critique dominant norms while also cultivating pleasure, solidarity, resilience, and belonging. Importantly, camp does not deny suffering. Rather, it often allows people to confront suffering obliquely by rendering painful realities more speakable, relational, and survivable. Camp therefore, occupies a complicated relationship to seriousness, wherein it does not reject serious realities like illness, stigma, death, or discrimination. Instead, it refuses the assumption that seriousness must always appear through solemnity, emotional austerity, or professional restraint.

This distinction is especially important in the context of nursing, which operates through an aesthetic of seriousness. Within healthcare settings, we might take as evidence of nursing's commitment to seriousness its emphasis on clinical competence, ethical responsibility, and professionalism, but this aesthetic of seriousness is far from neutral. Professional comportment in nursing is historically rooted in cisgender, heterosexual, white, upper‐middle‐class femininity and can be traced back to Florence Nightingale through generations of professionalization that positioned moral purity, emotional restraint, and decorum as markers of legitimacy (Power et al. 2025; Schuessler Poplin 1994). Seriousness has become so naturalized that alternative forms of communicating, dressing, relating, joking, grieving, or embodying care appear inherently unprofessional, or, at least, a degradation of professional legitimacy.

Camp becomes disruptive because it exposes supposed natural professional norms as performance. Drawing on Butler (1999) theorization of gender performativity, camp reveals that professionalism, much like gender, is maintained through repeated acts that create the illusion of coherence and inevitability. The ‘serious nurse’ is not a naturally occurring identity but a socially regulated performance produced through expectations regarding speech, dress, comportment, emotional expression, and embodiment. Because professionalism relies on appearing stable and natural, camp unsettles it by foregrounding theatricality. In doing so, camp exposes how institutional norms depend upon excluding certain bodies, affects, and expressions in order to maintain authority.

This dynamic helps explain why queer embodiment and camp aesthetics are often interpreted as incompatible with professional nursing spaces. When particular behaviors are prohibited or problematic in certain spaces, it is often because the very concept of ‘the space’ relies on the exclusion of certain forms of representation (Butler 2000). Viewed this way, camp is not a frivolous accessory to care, but rather a theoretical and practical method for reworking the terms of professionalism. Camp expands which bodies and expressions can inhabit the role of ‘nurse’, enabling modes of humor, intimacy, and irreverence that resist disciplinary demands, and opens new relational forms that resonate with marginalized communities. Camp thus becomes a queer framework for reimagining nursing practice itself.

For queer and trans nurses, this aesthetic of cisheteronormative professionalism is not only exclusionary but actively harmful (Röndahl 2011; Ziegler et al. 2025); it pressures them to suppress aspects of their identity, communication style, and relationality. Suppression of cultural practices could contribute to burnout (Shah et al. 2021) or otherwise support authentic practice, community rapport, and resilience. The enforced seriousness of nursing thus operates as a mechanism of cisheteronormative discipline rather than a universal standard of care, limiting both who feels welcome in the profession and which ways of caring are considered legitimate. Recognizing seriousness as a culturally produced aesthetic that one must perform opens the possibility of alternative modes of professionalism that can meet clinical responsibilities while refusing the normative constraints that stifle joy, authenticity, and queer presence.

Drag represents one particularly visible enactment of camp within queer culture, though the two are not synonymous. Drag involves the stylized performance of gender through costume, makeup, gesture, embodiment, and theatrical presentation. Camp frequently animates drag through exaggeration, parody, irony, and excess, but drag can emerge outside of formal drag performance. Drag nurses are not just spectacle, but also a broader possibility of nursing care that can incorporate theatricality, joy, irony, and queer relationality without sacrificing ethical seriousness or professional legitimacy.

This distinction clarifies why camp should not be confused with mockery. Critics may interpret drag or camp as degrading to professions or individuals because they misread exaggeration as ridicule. Camp often operates through affectionate overidentification rather than contempt (Sontag 1964). By exaggerating the figure of the nurse, drag nurses reveal and inhabit the symbolic power associated with nursing care (Butler 1999). Their performances do not necessarily reject nursing identity; rather, they reinhabit it differently. In doing so, they expose the limits of cisheteronormative professionalism while creating new possibilities for culturally congruent care.

This theoretical framing helps clarify why certain historical and contemporary drag nurses offer more than entertainment, satire, and theatricality in their work. They model an alternative camp‐inflected nursing practice that fosters connection rather than distance. The following sections build on this framework to examine how camp operates historically and contemporarily through nurses who mobilize drag as a form of community care.

### 1.2 Bobbi Campbell and Camp as Community Care

Bobbi Campbell's work as Sister Florence Nightmare RN did not emerge in isolation; it developed within broader queer networks of activism, mutual aid, performance, and community care that became vital during the early years of the HIV/AIDS pandemic. Bobbi was a member of the Sisters of Perpetual Indulgence, an eclectic order of queer and trans people who dress in modified nun habits and vivaciously dedicate their time and resources to community service, health education, fundraising for AIDS research and care, and promoting human wellbeing. Their mission is ‘to promulgate universal joy and to expiate stigmatic guilt’ and ‘helping others through humor and hard work’ (Glenn 2003; The sisters of perpetual indulgence 2001, p. 1).

The Sisters do not simply perform drag as entertainment, nor do they understand themselves as parodying Catholic nuns. They appropriate nun garb, practices, and institutional structures, thereby embodying the role of ‘nun’ to signal to the public that they fill that role despite unusual embodiment. Importantly, the Sisters reject the claim that they are drag queens or are drag nun caricatures, arguing that they are not dressing in nun drag to perform for entertainment, but rather to appropriate the role of the nun to ‘minister to the spiritual need of [their] community’ (McClelland 1997; Glenn 2003). They are consecrated as nuns for life and have religious and spiritual obligations arising from their lifelong commitment to service and activism. The Sisters *are* nuns. Their habits are not mere costumes; they are sacred, hard‐earned uniforms (Glenn 2003). Their vows are to be upheld irrespective of their outfit or setting. They embody nuns and fulfill the role of the nun: ‘visit the sick, feed the hungry, and generally service the community’ (The Antonio Sisters 2017, p. 2). Drag queens serve a different, at times overlapping, role with a primary focus on entertainment. The Sisters fulfill an important community role without sacrificing the fun drag queens bring, but they use it as a tool to advance their sociopolitical agenda to improve the lives of the queer/trans communities they care about (Glenn 2003). They are camp to the highest degree but do more than drag performances; they are satirical with something to say *and* something to do; they are mouthy with a clear message of community care.

The Sisters were/are not a nursing intervention, and I don't mean to impose professional nursing language onto practices they may understand differently. Rather, the Sisters operate in ways deeply resonant with nursing interventions, particularly health education, stigma reduction, advocacy, emotional support, and community‐based care. Their work reveals how camp can facilitate forms of relationality and public health engagement that conventional professionalism often struggles to achieve. They expose the performative nature of institutional authority, morality, and gendered respectability while simultaneously reinhabiting those symbols for queer community care. Their performances are affectionate and confrontational at once: playful without being unserious. In this way, camp allows the Sisters to interrupt shame and stigma through deliberate excess while creating visible public forms of queer solidarity rather than expected invisibility.

This was a particularly significant act during the HIV/AIDS crisis. In the early 1980s, HIV/AIDS was frequently ignored or dismissed by governments, healthcare systems, and broader society because it disproportionately affected gay men and other marginalized populations. Public discourse surrounding the disease was saturated with homophobia, fear, moral panic, and misinformation. Many healthcare institutions were ill‐equipped or outright unwilling to provide care. Queer communities often relied on mutual aid to survive. Under these conditions, modes of ‘serious’ public health communication often failed to reach those most vulnerable, or failed to do so in ways that acknowledge the lived realities of queer communities. Camp became one way of making conversations about these serious topics more emotionally survivable and culturally accessible.

In this context, the Sisters of Perpetual Indulgence supported Sister Florence Nightmare RN (Bobbi), Sister Roz Erection (Baruch Golden), and other Sisters in their development and distribution of a safer sex pamphlet aimed to reduce the spread of what would later be identified as HIV among gay men (The Sisters of Perpetual Indulgence 1982). Bobbi and Baruch were public health nurses who mobilized nursing knowledge through camp aesthetics. This pamphlet, entitled *Play Fair!*, is a raunchy, campy, and evidence‐based practical guide for gay men engaging in sexual activity.

**Image 1:**
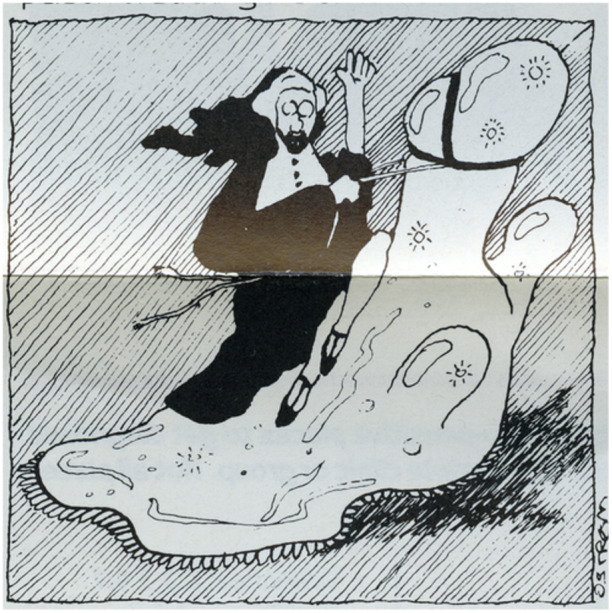
Courtesy of UCSF archives and special collections.

*Play Fair!* starts with a farcical story about the signs of ‘sexually transmitted diseases’ that Mother Superior recently identified among the Sisters. Alongside detailed information about sexually transmitted infections, hygiene, condom use, practices to avoid (e.g., anonymous sex), and local clinics offering non‐discriminatory treatment, readers encounter cartoons of nuns in various comical situations. One image depicts a nun riding an amoeba side‐saddle like a horse next to information about intestinal parasites (see Image 1). Another shows a Sister looks aghast at a urinal as her urine goes up in flames and smoke while the reader learns about gonorrhea. The pamphlet is unapologetically camp and oscillates constantly between raunchy comedy and clinical education.

The camp aesthetic serves multiple functions. First, they break up dense text that describes stigmatized infections and their medical management. More importantly, they are culturally humorous, intended to challenge the stigma associated with sexually transmitted infections through laughter and shared social reference points. The Sister nurses understood their audience and their desired outcome for this pamphlet. They used the cultural practice of camp as a vehicle to entice gay men to learn about the uptick in sexually transmitted infections, including symptoms of a new and mysterious [suspected] infection that was associated with cancer, pneumonia, and death.

Though camp did not mean that HIV/AIDS or sexually transmitted infections were being treated unseriously. Bobbi was actively living with HIV/AIDS while performing as Florence Nightmare RN, and would later die from complications related to AIDS (Lipsky 2021). The seriousness of terminal illness is often at the forefront of people's minds, and Bobbi is known to have had bouts of depression after his diagnosis (France 2016). The gravity of the situation was compounded by his knowledge of healthcare, lack of treatment options, understanding of emerging data that showed discouraging mortality rates (France 2016), and the stigma that accompanied an AIDS diagnosis (Wright 2013). Yet rather than retreat into the emotionally restrained posture expected of professional seriousness, Bobbi used camp to make unbearable realities discussable within his community.

This combination is compounded further by Bobbi's public education campaign about the ‘gay cancer’ Kaposi's sarcoma, in which he used images of his own body to educate others about the disease (Lipsky 2021), literally indicating to onlookers that he had skin in the game. Such acts disrupted conventional expectations of professional distance between nurse and patient, caregiver and suffer. Bobbi refused the illusion that nurses exist outside of vulnerability, illness, or embodiment. His willingness to place his own body visibly within the public health discourse transformed education into a radically personal, relational, and political act. His message was not only informational but communal. He challenged the silence and shame around Kaposi's sarcoma or ‘gay cancer’ at a historical moment when many institutions preferred queer suffering to remain unseen.

Bobbi's drag was not an escape from nursing professionalism, nor a parody of nursing. It expanded the restrictive confines of cisheteronormative seriousness by demonstrating that care could be theatrical, humorous, vulnerable, politically confrontational, and culturally specific while remaining ethical and clinically meaningful. His work illustrates how camp can function as a mode of nursing intervention that is used to create spaces for collective survival under conditions of abandonment and fear.

Bobbi and the Sisters reveal something broader about nursing aesthetics and queer care. Humor and theatricality are not necessarily oppositional to serious healthcare work. In some contexts, camp may allow communities to hear, discuss, and engage life‐threatening realities more effectively than conventional professional discourse alone. Rather, it creates relational openings through which care becomes emotionally survivable, politically resistant, and culturally meaningful.

### 1.3 The Ongoing Legacy of the Drag Nurse

Camp‐inflected nursing did not end with Sister Florence Nightmare RN. Across decades and geographic contexts, drag nurses have used camp to do what nursing has always done: promote health, build trust, and reduce harm. They do so through culturally resonant queer aesthetics. These examples show that camp nursing is not reducible to spectacle or entertainment, but rather it functions as a mode of relational care that expands how nursing interventions are communicated and experienced.

**Image 2:**
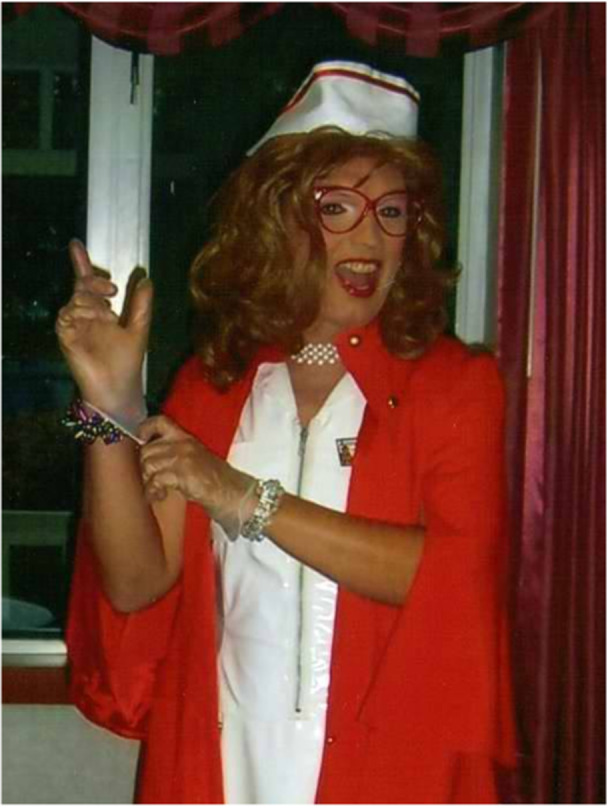
Image courtesy of Bob Bolyard.

In Burlington, VT, where I currently reside, the House of Lemay is a long‐established drag troupe that included a local nurse with the stage name Nurse Anne Thracks, pronounced ‘Anthrax’ (see Image 2). Nurse Anne Thracks regularly performed at the troupe's annual Winter is a Drag Ball, where she sang an upbeat holiday song about the importance of the flu vaccine, distributed hand sanitizer and tissues, and administered vaccines while working as a nurse dressed in full drag. She foregrounded her nursing expertise in her drag persona, rather than distancing herself from it. This decision established a relationship with her audience and turned a mundane, painful health task into a spectacle.

To paraphrase one of her patients, Nurse Anne Thracks single‐handedly raised awareness, provided education and primary prevention care/resources, and distracted people from a task they would have otherwise skipped or reluctantly attended by turning it into a joyful and deliciously queer experience. Her patients relished her cheerfulness and often wanted more engagement with her in future health maintenance programs she hosted. Camp and drag were used to turn tedious care into a fun, or even pleasurable, experience, which encourages long‐term engagement with healthcare services. This is particularly crucial for populations who have historically been harmed by the healthcare system and are subsequently avoidant or fearful, such as the queer and trans community. Camp and drag were used to expand the cisheteronormative experience of healthcare to lovingly include queer and trans people in culturally congruent ways. Nurse Anne Thracks began breaking down disparities resulting from queer and trans people's fear of discrimination in healthcare because they saw queer camp represented in their own care. While she is now retired, her legacy in the Burlington queer community lives on.

Contemporary drag nursing has also expanded into digital spaces through social media and online performance. Mandy Mango is a drag performer on RuPaul's Drag Race Season 18. According to her social media, she has conducted edutainment sessions about inclusive sexual education with her credentials as an HIV/AIDS Certified Registered Nurse (ACRN), as highlighted as her cheekbones (Mandy Mango @mandyy.mango 2025). Using her credentials lends her the social capital associated with being a nurse (i.e., the most trusted profession) and buys trust from her audience, which encourages engagement with health education materials.

In one performance, Mandy dresses up in a saucy Halloween‐style nurse's costume—white cap and all—and sings about inclusive safer‐sex practices during the COVID‐19 pandemic (Mandy Mango @mandyy.mango 2020 *)*. In this instance, a drag nurse is educating their community through evidence‐based, practical education to prevent the spread of communicable diseases, facilitate meaningful and inclusive sexual experiences, and build trust within a community that was historically discriminated against in healthcare settings. This is not unlike *Play Fair!* which also used camp and drag to further public health knowledge and promote the well‐being of people engaging in sexual activity with partners during a different pandemic. Mango's reach on social media is potentially larger, as she can quickly spread information with her community. She does so in the same way Sister Florence Nightmare and Nurse Anne Thracks did before her: a nurse dressed as an over‐the‐top caricature of a nurse with a cheeky sense of humor and desire to provide care to their community through action.

Sister Florence Nightmare, Nurse Anne Thracks, and Mandy Mango do not mock or parody nurses in the same way the Sisters of Perpetual Indulgence do not ridicule nuns; they *are* nurses *performing* nursing interventions by reinhabiting the recognizable figure of ‘the nurse’ through camp. It transforms health education from top‐down instruction into relational engagement. It creates opportunity for joy and collective participation in contexts shaped by illness, fear, or institutional neglect. Most importantly, camp demonstrates that effective nursing care does not require emotional austerity or rigid adherence to cisheteronormative aesthetics of professionalism.

I am certain there are other drag nurses doing the same vital work across the globe, but many nurses may not want to perform camp to the same extreme as the drag nurse. Camp can be more subtle in everyday nursing settings. Pediatric nurses may use theatrical humor and exaggerated playfulness to reduce children's anxiety during frightening procedures. Hospice nurses may incorporate irony, humor, and relational wit into conversations about death and grief to preserve dignity and emotional connection. Sexual health educators may intentionally disrupt clinical awkwardness through playful language, flamboyant visual materials, or culturally specific humor. Mental health and community health nurses may similarly cultivate camp sensibilities through aesthetic warmth, expressive communication, or deliberate resistance to emotionally sterile forms of institutional interaction. While camp may not always be universally appropriate, nurses can gauge their relationships with the people they care for to determine when to use camp‐inflected interventions to strengthen those relationships.

The ongoing legacy of the drag nurse and other camp nurses reveals that it is not merely about making care fun. Aesthetics shape trust, belonging, communication, and relational possibility within clinical and community settings. By disrupting the narrow confines of cisheteronormative professionalism, camp opens space for forms of care that are emotionally accessible, culturally responsive, politically conscious, and authentically human.

### 1.4 Camp as a Corrective and a Catalyst

Camp nursing unsettles the assumption that ethical care must appear emotionally restrained, aesthetically neutral, or institutionally respectable in order to remain legitimate. Users of camp take products of the status quo, and occupy those objects in innovative and irreverent ways without defiling the object (Sontag 1964). In all its forms, camp becomes a method for critiquing the systems that reproduce injustice using wit, parody, and humor to puncture the seriousness that often masks oppression. Upholding the status quo is therefore in direct competition with the purpose of camp and drag. By foregrounding camp, professionalism is exposed as a culturally specific performance rather than a universal expression of competence. In doing so, camp becomes both corrective and a catalyst: corrective in its challenge to restrictive norms governing who can appear recognizable as a ‘professional nurse’ and a catalyst in its ability to demand innovation and emphasize inherent dignity in culturally responsive, relational care.

While camp‐inflected nursing and drag nurses may unsettle those who equate professionalism with cisheteronormative restraint, their benefits are undeniable. For over forty years in the United States, drag nurses like Sister Florence Nightmare, RN, have demonstrated measurable contributions to community health through direct, culturally resonant nursing intervention. At the same time, camp nursing is not universally appropriate, nor should it be romanticized as inherently liberatory. Not every patient desires theatricality or humor in healthcare encounters, and camp can itself reproduce exclusionary dynamics depending on how it's mobilized. My argument is to challenge the assumption that professionalism has only one legitimate aesthetic expression. Camp expands the repertoire of what ethical nursing can look and feel like, without abandoning commitments to competence, accountability, and patient wellbeing.

Camp has implications for nursing ethics. The American Nurses Association's Nursing Code of Ethics (American Nurses Association 2025) states, ‘the nurse has moral duties to self as a person of inherent dignity and worth, including… authenticity of self at work’ (American Nurses Association 2025). There is an implicit conflict between the assertion that nurses have a moral duty to themselves to be authentic at work and the expectation that they perform according to cisheteronormative definitions of seriousness or professionalism. For queer and trans nurses especially, authentic nursing practice may involve integration of camp into patient education, advocacy, and care. For some, authenticity is embodying a drag nurse and edutaining community members in a performance. If the profession is to uphold the American Nurses Association's Code of Ethics, it must interrogate the assumptions about professionalism and seriousness embedded in curricula, public spaces, and policies that implicitly or explicitly exclude different modes of expression. With camp‐inflected nursing, a wide range of interventions that were dismissed or delegitimized under the cisheteronormative model of seriousness become newly visible, possible, and powerful. As Sontag (1964) reminds readers, camp is not the exclusive property of queer/trans people, and many marginalized groups intuitively relate to camp's ‘life‐as‐theater’ (Sontag 1964, p. 12), integrating their brand of camp into their labor, resistance, and care. Camp nursing is not simply aesthetic. It is ethical.

Critics may see camp‐inflected nursing and the drag nurse as degradation to the profession because such practices disrupt long‐standing associations between professionalism, seriousness, and restraint. These concerns often emerge from respectability politics that treat flamboyance, humor, queer embodiment, or theatricality as incompatible with clinical competence. Yet the examples throughout this paper demonstrate otherwise. Sister Florence Nightmare RN, Nurse Anne Thracks, and Mandy Mango all mobilized nursing expertise through camp aesthetics while facilitating public health education, reducing stigma, cultivating trust, and supporting community wellbeing. Their work does not diminish nursing practice; it expands its communicative and relational possibilities.

Importantly, drag nurses are not merely pretending to be nurses while dressing in costume, like the Sisters of Perpetual Indulgence misread by Catholic Nuns (Glenn 2003). Just as the Sisters structured their order around the life of nuns, camp and drag nurses take the culturally recognizable figure of the ‘nurse’ by adapting names (Florence Nightingale), wearing garb (scrubs, white dresses, and caps), and using their real credentials (RN, ACRN) and reinhabit it for the purposes of care. They *are* nurses *doing* nursing. By introducing playfulness where traditional norms expected stoicism, they expose the rigidity of professional expectations while acknowledging the gravity of nursing work and refusing to allow seriousness to become synonymous with emotional rigidity or depersonalization. That is, camp does not make the act of drag nursing any less crucial or meaningful than traditional ‘serious’ nursing. Instead, it shows that campy nursing is innovative, generative, and can expand to create space for historically marginalized individuals previously excluded by the cisheteronormative model of nursing practice.

Camp therefore, functions not as a rejection of nursing, but as a challenge to the narrowness of its current form. By embracing camp in nursing ontology and epistemology, nurses can disrupt the politics of seriousness that suppress joy in the name of institutional legitimacy. Camp nursing enables an aesthetic practice that fully acknowledges the gravity of nursing practice while refusing to allow seriousness to become synonymous with emotional austerity. Camp‐inflected nurses can look at the drag nurses that preceded them not as outliers, but as visionaries who reveal what nursing can become when we refuse the narrow confines of cisheteronormative professionalism. The work of the drag nurse shows that camp does not undermine the gravity of nursing responsibilities, but rather it clears space for joy, connection, cultural relevance, and collective resilience in the face of suffering.

## 2 Conclusion

Recognizing camp as a legitimate nursing aesthetic allows the profession to expand the boundaries of what counts as professional, ethical, or therapeutic, whether through subtle playfulness, bold drag performances, or anything in between. This reimagining does more than make nursing more hospitable for queer and trans practitioners and patients. It strengthens the discipline by honoring authenticity, relationality, and sociopolitical wellness as integral to care. In the end, camp is not a distraction from nursing, but a generative force within it, inviting the profession to move beyond narrow definitions of professional legitimacy and seriousness towards a practice infused with humanity, humor, and liberation.

## Funding

The author has nothing to report.

## Ethics Statement

The author has nothing to report.

## Conflicts of Interest

The author declares no conflicts of interest.

## Data Availability

Data sharing is not applicable to this article as no datasets were generated or analyzed during the current study.
